# Individual Subject Classification of Mixed Dementia from Pure Subcortical Vascular Dementia Based on Subcortical Shape Analysis

**DOI:** 10.1371/journal.pone.0075602

**Published:** 2013-10-10

**Authors:** Hee Jin Kim, Jeonghun Kim, Hanna Cho, Byoung Seok Ye, Cindy W. Yoon, Young Noh, Geon Ha Kim, Jae Hong Lee, Jae Seung Kim, Yearn Seong Choe, Kyung-Han Lee, Chang-Hun Kim, Sang Won Seo, Michael W. Weiner, Duk L. Na, Joon-Kyung Seong

**Affiliations:** 1 Department of Neurology, Samsung Medical Center, Sungkyunkwan University School of Medicine, Seoul, Korea; 2 Department of Computer and Radio Communications Engineering, Korea University, Seoul, Korea; 3 Department of Neurology, Inha University College of Medicine, Incheon, Korea; 4 Department of Neurology, Gachon University Gil Medical Center, Incheon, Korea; 5 Department of Neurology, Ewha Womans University Mokdong Hospital, Ewha Womans University School of Medicine, Seoul, Korea; 6 Department of Neurology, University of Ulsan College of Medicine, Asan Medical Center, Seoul, Korea; 7 Department of Nuclear Medicine, University of Ulsan College of Medicine, Asan Medical Center, Seoul, Korea; 8 Department of Nuclear Medicine, Samsung Medical Center, Sungkyunkwan University School of Medicine, Seoul, Korea; 9 University of California San Francisco, San Francisco, California, United States of America; 10 Center for Imaging of Neurodegenerative Diseases, Department of Veterans Affairs Medical Center, San Francisco, California, United States of America; 11 Department of Biomedical Engineering, Korea University, Seoul, Korea; Institution of Automation, CAS, China

## Abstract

Subcortical vascular dementia (SVaD), one of common causes of dementia, has concomitant Alzheimer's disease (AD) pathology in over 30%, termed “mixed dementia”. Identifying mixed dementia from SVaD is important because potential amyloid-targeted therapies may be effective for treatment in mixed dementia. The purpose of this study was to discriminate mixed dementia from pure SVaD using magnetic resonance imaging (MRI). We measured brain amyloid deposition using the 11C-Pittsburgh compound B positron emission tomography (PiB-PET) in 68 patients with SVaD. A PiB retention ratio greater than 1.5 was considered PiB(+). Hippocampal and amygdalar shape were used in the incremental learning method to discriminate mixed dementia from pure SVaD because these structures are known to be prominently involved by AD pathologies. Among 68 patients, 23 (33.8%) patients were positive for PiB binding. With use of hippocampal shape analysis alone, PiB(+) SVaD could be discriminated from PiB(-) SVaD with 77.9% accuracy (95.7% sensitivity and 68.9% specificity). With use of amygdalar shape, the discrimination accuracy was 75.0% (87.0% sensitivity and 68.9% specificity). When hippocampal and amygdalar shape were analyzed together, accuracy increased to 82.4% (95.7% sensitivity and 75.6% specificity). An incremental learning method using hippocampal and amygdalar shape distinguishes mixed dementia from pure SVaD. Furthermore, our results suggest that amyloid pathology and vascular pathology have different effects on the shape of the hippocampus and amygdala.

## Introduction

Subcortical vascular dementia (SVaD) is one of common causes of dementia [1], and is characterized by extensive white matter hyperintensities (WMH) and multiple lacunes. Pathological studies have demonstrated that some patients who have been clinically diagnosed with SVaD also have co-associated Alzheimer's disease (AD) pathologies [2], [3]. Thus, the concept of mixed dementia has emerged. A recent study using the Pittsburgh compound B positron emission tomography (PiB-PET) [4], a sensitive method of detecting amyloid in fibrillary form, has shown that about 30% of patients who have been clinically diagnosed with SVaD present with significant amyloid burden, indicating that these patients have mixed dementia [5].

There is a need to detect the presence of AD pathology in subjects with clinical SVaD. Although there is considerable overlap, mixed dementia and pure SVaD differ in terms of clinical phenotype and treatment. A previous study indicated that, clinically, patients with mixed dementia were older and performed worse on memory tests than those with pure SVaD [5]. In regards to treatment, patients with mixed dementia could potentially benefit from future amyloid-targeted therapies while management of vascular risk factors and antiplatelet therapies may be more focused in patients with pure SVaD.

Although amyloid PET and cerebrospinal fluid (CSF) studies can detect concomitant amyloid burden in the brain, these methods are limited in clinical practice in the following ways. Primarily, the half-life of 11C, a radio ligand of PiB-PET, is only 20–30 minutes; while another amyloid PET, Amyvid (using amyloid ligand 18F), has been approved by the United States Food and Drug Administration [6], it is expensive and not yet widely available. An alternative method can detect low amyloid beta 42 and high tau levels in CSF [7]. However, CSF studies are invasive and results can vary among laboratories. Thus, it is important to diagnose mixed dementia patients using widely available data such as structural magnetic resonance imaging (MRI), before using expensive or invasive tools.

It is well known that the medial temporal regions including hippocampus and amygdala are structures that are atrophied in patients with AD [8]. Pathological studies suggested that AD pathology is involved in hippocampus and amygdala even in the early stage [9]. Thus imaging analysis of these structures has recently been used in research of AD [10]–[12]. Therefore it can be assumed that concomitant AD pathology in SVaD may also give rise to atrophy in the hippocampus and amygdala. Indeed, a previous study has shown that by using a visual rating, mixed dementia patients have demonstrated more severe hippocampal atrophy than pure SVaD patients [5].

Characteristics of mixed dementia that differ from pure SVaD have been proposed [5]. However, an individual subject classification model rather than a group analysis would provide more practical information to individual patients. Numerous classification models have been suggested to discern between certain groups in clinical practice. In general, clinical and imaging data have been used to classify individual patients for diagnosis [13], [14] or for predicting disease progression [15], [16]. Most classification models have been based on the Alzheimer's Disease Neuroimaging Initiative dataset, and thus have focused on AD dementia and its prodromal stage [14], [15], [17]. To our knowledge, there have been no previous studies that have provided an individual subject classification model to distinguish mixed dementia from pure SVaD.

In this study, we aimed to discriminate mixed dementia from pure SVaD by employing surface-based subcortical shape analysis methods. Our methods combine MRI information that is typical for AD, including changes in hippocampal and amygdalar shape. Both hippocampal and amygdalar shape information were combined to construct the feature vector in high-dimensional space (i.e., more than 8000 dimensions). In order to effectively reduce the feature data, we used a manifold harmonic transform and principal component analysis (PCA), which facilitates classification with high accuracy [16]. Furthermore, an incremental learning method [16] was used to train our classifier. MR images are usually obtained during disease diagnosis and, thus, the volume of data increases steadily, which justifies our classification method based on incremental learning.

## Methods

### Ethics Statement

The study was approved by the Institutional Review Board of the Samsung Medical Center. We obtained written informed consent from all the participants. Structured written consent procedures were used by research staff when approaching participants with cognitive impairment. The assent of “next of kin” was required for participation of people with cognitive impairment who were unable to provide informed consent.

### Participants

From September 2008 to August 2011 we prospectively recruited 98 patients with SVaD. SVaD was determined using the diagnostic criteria for vascular dementia as defined by the Diagnostic and Statistical Manual of Mental Disorders–Fourth Edition (DSM-IV). Patients were evaluated in a clinical interview and neurological and neuropsychological examinations as previously described [18]. All SVaD patients had a significant ischemia on their MRI scans, which was defined as having a cap or band of ≥10 mm as well as a deep white matter lesion of ≥25 mm, as modified from the Fazekas ischemia criteria [19]. We excluded patients with territory infarctions and those with high signal abnormalities on the MRI due to radiation injury, multiple sclerosis, vasculitis, or leukodystrophy. We also excluded patients who met Diagnostic and Statistical Manual of Mental Disorders, Fourth Edition criteria for psychotic disorder or mood disorder such as schizophrenia or major depressive disorder. In order to exclude secondary causes of cognitive deficits, all patients completed laboratory tests including a complete blood count, blood chemistry, vitamin B_12_/folate, syphilis serology, and thyroid function tests. Brain MRI scanning confirmed the absence of structural lesions including territorial cerebral infarction, brain tumor, hippocampal sclerosis, and vascular malformation.

Of the 98 SVaD patients, 70 patients completed a [11C] PiB-PET scan. During the process of imaging analysis, an error occurred in two patients. Thus a total of 68 patients were analyzed in this study.

### [11C] PiB-PET

All 70 patients completed the [11C] PiB-PET scan at Samsung Medical Center or Asan Medical Center. All subjects completed the same type of PETscan with a Discovery STe PET/computed tomography scanner (GE Medical Systems, Milwaukee, WI, USA) in a 3-D scanning mode that examined 35 slices of 4.25-mm thickness that spanned the entire brain. The 11C-PiB was injected into an antecubital vein as a bolus with a mean dose of 420 MBq (range, 259e550 MBq). A computed tomography scan was performed for attenuation correction at 60 minutes after the injection. A 30-minute emission static PET scan was then initiated [5].

PiB-PET images were coregistered to individual MRI scans, which were normalized to a T1-weighted MRI template. Using these parameters, MRI-coregistered PiB-PET images were normalized to the MRI template. The quantitative regional values of PiB retention on the spatially normalized PiB images were obtained by an automated volume of interest (VOI) analysis using the automated anatomical labeling atlas. Data processing was performed using the SPM version 5 (SPM5) under Matlab 6.5 (Mathworks, Natick, MA, USA). To measure PiB retention, we used the cerebral cortical region to cerebellum uptake ratio which is identical to the standardized uptake value ratios (SUVRs). The cerebellum was used as a reference region because it did not show group differences. We selected 28 cortical VOIs from left and right hemispheres using the automated anatomical labeling atlas. The cerebral cortical VOIs which were chosen for this study consisted of bilateral frontal (superior and middle frontal gyri, medial part of the superior frontal gyrus, opercular part of the inferior frontal gyrus, triangular part of the inferior frontal gyrus, supplementary motor area, orbital part of the superior, middle, and inferior orbital frontal gyri, rectus, and olfactory cortex), posterior cingulate gyri, parietal (superior and inferior parietal, supramarginal and angular gyri, and precuneus), lateral temporal (superior, middle, and inferior temporal gyri, and Heschl gyri), and occipital (superior, middle, and inferior occipital gyri, cuneus, calcarine fissure, and lingual and fusiform gyri). Regional cerebral cortical SUVRs were calculated by dividing each cortical VOI's standardized uptake value by mean standardized uptake value of the cerebellar cortex. Global PiB retention ratio was calculated from the volume-weighted average SUVR of 28 bilateral cerebral cortical VOIs [5]. A PiB retention ratio greater than 1.5 was considered PiB(+).

### MR imaging techniques

T2, T1, FLAIR and T2 Fast Field Echo (FFE) MR images were acquired from 70 subjects with SVaD at the Samsung Medical Center using the same 3.0 T MRI scanner (Philips 3.0T Achieva). In all patients, these images were obtained in one session and all MR images were obtained in the same orientation and slice positions. FLAIR MR images were acquired in the axial plane with the following parameters: axial slice thickness of 2 mm, no gap; repetition time (TR) of 11000.0 ms; echo time (TE) of 125.0 ms; flip angle of 90°; and matrix size of 512x512 pixels. T2 Fast Field Echo (FFE) images were obtained using the following parameters: axial slice thickness of 5.0 mm; inter-slice thickness of 2 mm; TR, 669 ms; TE 16 ms; flip angle of 18°; and matrix size of 560×560 pixels.

**Figure 1:**
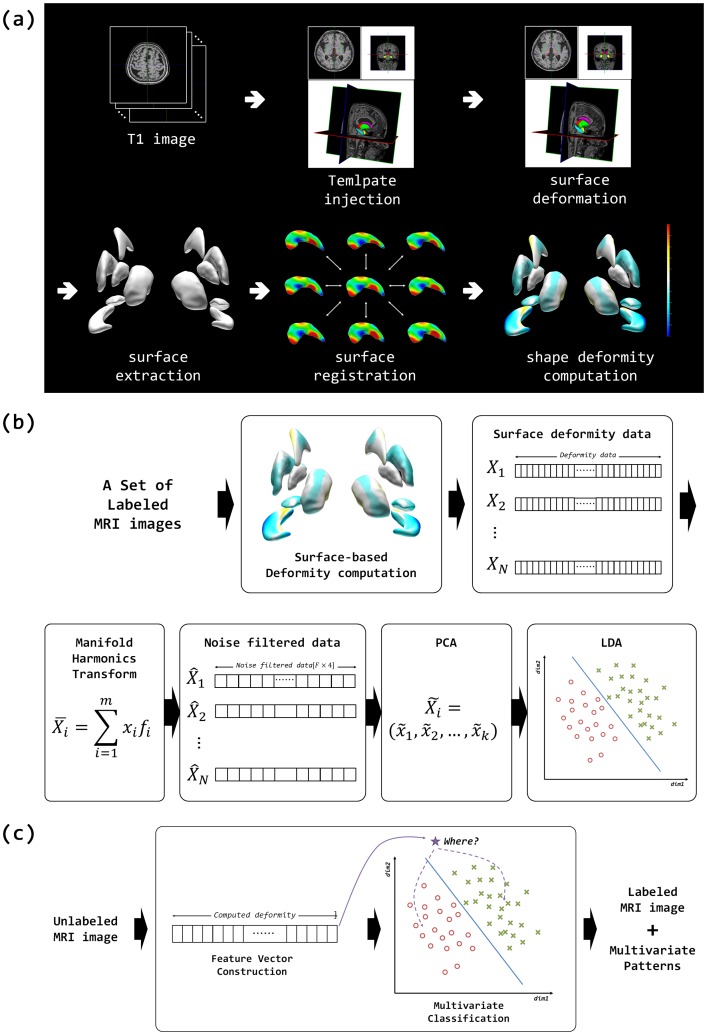
Overview of the proposed classification method: (a) subcortical shape analysis, (b) multivariate classifier training, and (c) individual subject classification.

Our method aimed to classify mixed dementia from pure SVaD by employing the following three steps: subcortical shape analysis, multivariate classifier learning, and individual subject classification. Figure 1 presents an overview of our method. Details of each step are described in the following sections.

### Shape analysis of subcortical structures

We performed shape analysis of subcortical structures by measuring relative deformation of subcortical surface meshes against the template mesh. The shape analysis process consists of four steps: volume parcellation, surface extraction, registration, and surface deformity computation (see Figure 1[a]). The first step obtains the anatomical parcellations of human subcortical structures from the T1 images of each patient using the FreeSurfer software package (Version 5.0, Athinoula A. Martinos Center at the Massachusetts General Hospital, Harvard Medical School; http://www.surfer.nmr.mgh.harvard.edu/). The parcellated images were then transformed to native anatomical space for surface extraction. The second step extracts surface meshes of the subcortical structures for each subject by deforming the template surface models. Specifically, we utilized the subcortical shape atlas models [20] as a template surface and used the Laplacian-based surface deformation method [21], [22] in order to extract the subcortical surfaces of each subject. The third step involved surface registration in order to establish the vertex correspondence of subcortical surface meshes across the sample. We used the surface registration method developed by Cho et al. (2012) for accurate registration. Finally, surface deformity of each subcortical structure was measured against the template surface model by calculating the vertex-wise spatial displacement along its outward normal direction. In order to correct the brain size effect when computing the shape deformity data, each subcortical surface mesh was transformed from native anatomical space to the template space using the inverse of the registration mapping.

In order to quantitatively validate our method for subcortical surface construction, we performed a comparison test using manually-delineated hippocampal volumes. An expert neuroanatomist manually delineated hippocampal volumes of 20 healthy subjects from the T1 images slice-by-slice using version 4.2.2 of 3D Slicer [23]. For the same set of healthy subjects, we extracted hippocampal surface meshes using our surface construction method described above. The hippocampal volume images were then reverse-engineered from the reconstructed surface meshes by superimposing the meshes on top of the input MR images. We traversed every voxel of the input MR images, and tested if the voxel is inside/outside the hippocampal surface mesh to construct the hippocampal volume images. Finally, the volume overlap was measured between the reverse-engineered volumes and the manually delineated ones: we observed that the two hippocampal volumes were overlapped for more than 90% of the manually-parcellated volumes. This result shows that our Laplacian-based surface modeling method [24], [25] accurately constructs subcortical surface meshes from the automatically-parcellated subcortical volumes.

### Multivariate classification using linear discriminant analysis

Patterns of subcortical structure atrophies using MR structural imaging have been utilized as significant biomarkers for the diagnosis of Alzheimer's disease (AD). In this study, we adopted the incremental learning method proposed by Cho et al. (2012) to analyze multivariate patterns of subcortical shape change that are particularly useful in discriminating mixed dementia from pure SVaD. The multivariate pattern analysis (MVPA) identifies regions where spatially distributed patterns of subcortical shape differences are evident between the two groups. In order to train the multivariate classifier, we used linear discriminant analysis (LDA) in combination with principal component analysis (PCA), which was developed in machine learning to classify items based on a linear separation in high-dimensional feature space [26]–[28]. LDA provides not only statistical measures of classification accuracy, but also regional information about differences of two groups based on how accurately or poorly they can be discriminated with LDA.

Our multivariate pattern classification method consisted of two steps: classifier training and individual subject classification (see Figures 1[a] and [b]). The former step trained a classifier with labeled MR volumes. We represented the shape deformity data of subcortical structures for each subject in terms of their spatial frequency components by using the manifold harmonic transform. Specifically, for a surface mesh with 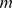 vertices, the shape deformity data is represented using a 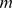-dimensional vector 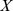. In our study, 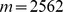 for the hippocampal and amygdale surface meshes. Then, the transformed vector 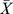 can be represented as 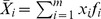, where \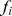 is the 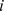
th eigenvector of the manifold harmonic transform. This step then filtered out high frequency components from the shape deformity data at vertices, which had been extracted from the MR volumes in order to remove noise. The resulting feature vector is now 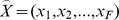, where 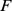 is a cut-off frequency: in our study, 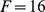, which was determined in a similar manner to our previous study [16]. We then used PCA to reduce the dimension of the feature space, which prevents the singularity problem in performing LDA. We empirically decided the dimension of the reduced feature space by setting the percentage of the total variance to 70%. Finally, the classifier was obtained by using LDA with the transformed training data 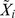, 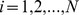 in the reduced PCA space. Among human subcortical structures, shape deformities of both hippocampi and the amygdala were used to represent high-dimensional features of brain atrophy since those structures have been well known to be substantially more vulnerable in AD.

The latter step classified unlabeled subjects by using an individual subject classifier. This classifier was initialized with the classifier trained in the previous step and incrementally updated. Given the MR volume of a subject, the feature vector representing the noise-filtered subcortical shape deformity data was acquired, as in classifier training. Finally, the classifier performed the multivariate classification using the high-dimensional feature vector.

The LDA analysis between PiB(+) and PiB(-) SVaD was completed using three different types of feature vectors: hippocampal shape deformity, amygdalar shape deformity, and their combination. For each type of feature vector, we measured classification accuracy and analyzed the multivariate patterns that showed discriminative regions. Since different types of feature vectors were given and standardized scores were not available, a principal component analysis was performed separately to reduce the number of dimensions to *N* eigenvectors, where *N* was the minimum number of components that accounted for at least 70% of the variance.

For assessment of classification performances, we performed a 10-fold, cross-validation procedure. We first trained three classifiers for the PiB(+) versus PiB(-) SVaD classification using the three different types of feature vectors. Specifically, for training a classifier, we used 90% of the patients (those patients were selected randomly from the total set of patients) as a training data set to train the LDA-based multivariate classifier. We then used the other 10% of the patients as test data for identifying their label and for extracting multivariate patterns of subcortical shape change. This process of training a classifier with 90% of the patients and testing on the other 10% of the patients was repeated 10 times until all patients had been used as test data at least once. Prediction accuracy, sensitivity, specificity, and discriminative regions were calculated.

In order to measure the prediction accuracy unbiased to a specific ordering of the patients in the data sets, we generated 500 random permutations of all the training patients for each of the training data for the three classifiers. For each permutation, we measured the accuracy of the classifier using the corresponding test patient data by incrementally updating the classifier; the training data for 10 patients were iteratively supplied at a time for incremental learning until all the training data were used. Whenever the classifier was updated with the training patients, all the test data were used to estimate the accuracy of the classifier. We then measured the accuracy of the classifier with respect to the number of used training data by averaging the results over all permutations.

Our learning method was based on both PCA and LDA, which train the classifier in an incremental manner. Specifically, whenever a new data set was obtained, our classification method trained the classifier incrementally with the newly obtained data set. In contrast, conventional classification methods train the classifier with an entire data set, including new data, in order to additively reflect the new data, which is not much in general. Since new data are obtained usually from the disease diagnosis stage, the volume of data increases steadily. Under these circumstances, our incremental learning-based classification method effectively addresses the problem of continued data acquisition at this stage. Please refer to our previous paper [16] for more details of the incremental learning-based classification method.

### Identification of the discriminative regions

In order to analyze regional information about the differences between PiB(+) and PiB(-) SVaD groups, we computed the distinguishing regions that contributed to classifying the two groups. LDA finds a separating axis w that maximally separates groups for learning a classifier. The value of the ith component of the vector w represents the contribution of the component to classification. That is, if the value of the ith component of w is zero, the ith element of every feature vector does not affect the classification result. Conversely, if the value is larger than the others, the classification result is more sensitive to the ith element than the other elements. Therefore, the analysis of w provides the discriminative region for classification. We visualized the axis w of LDA by converting it to a pair of vectors on the left and right atlas meshes. The w in the PCA space is first transformed to a vector in the feature space. The vector is then divided into two parts: frequency components for the left and right atlas meshes. These frequency components are finally transformed to two subcortical deformity vectors on the left and right atlas meshes, respectively. We divided these vectors by their magnitudes to obtain two unit vectors for visualization.

### Statistics

We compared the demographic and clinical data among the groups using Student *t* tests for continuous variables and Chi-square tests for dichotomous variables.

## Results

### Clinical results

As shown in Table 1, PiB(+) SVaD and PiB(-) SVaD patients showed significant differencies in age, number of lacunes, and Apolipoprotein E (APOE) genotype. Patients with PiB(+) SVaD were older, had fewer lacunes, and had more APOE4 carriers, as compared to PiB(-) SVaD patients.

**Table 1:** Clinical characteristics of patients with PiB(-) and PiB(+) SVaD.

	PiB(-) (n = 45)	PiB(+) (n = 23)	p
**Demographics**			
Age	71.9±7.4	78.1±4.7	<0.001
Gender, M:F	21:24	06:17	0.101
Education	8.3±4.8	9.1±5.5	0.523
**Risk factors**			
Hypertention	36 (80%)	18 (78.3%)	1.000
Diabetes	11 (24.4%)	6 (26.1%)	0.882
Hyperlipidemia	20 (44.4%)	6 (26.1%)	0.141
**Cognition**			
MMSE	21.4±4.7	18.7±4.9	0.036
CDR-SOB	6.0±3.8	6.5±3.8	0.61
**MRI markers**			
WMH volume, ml	41.9±15.6	45.5±22.9	0.447
Total lacune, n	21.1±18.3	7.0±6.9	<0.001
Intracranial volume	1381.4±134.3	1390.4±155.1	0.804
**APOE genotype**			
APOE4 carriers	9 (21.4%)	10 (43.5%)	0.062
**Medication**			
Antipsychotics	1 (2.2%)	2 (8.7%)	0.219
Antidepressant	5 (11.1%)	2 (8.7%)	0.756
Anxiolytics	4 (8.9%)	4 (17.4%)	0.303

Abbreviations: PiB, Pittsburgh compound B; MMSE, Mini-Mental State Examination; CDR-SOB, clinical dementia rating sum of boxes; WMH, white matter hyperintensities; APOE4, Apolipoprotein E epsiolon 4.

### Multivariate classification results

**Figure 2:**
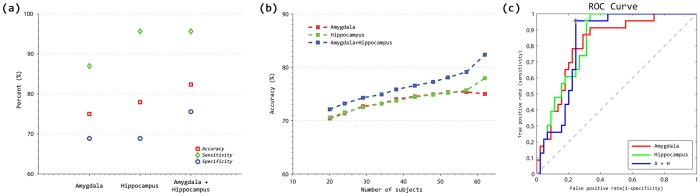
Accuracy of individual subject classifiers: (a) classifiers were trained using all entire training data and (b) the classifiers were trained in an incremental manner. For incremental learning, averaged accuracies of individual subject classifiers were shown with respect to the number of training subjects used. The average accuracy of each classifier tended to increase with the number of training subjects used. (c) the ROC curves of each classifier are shown.

To assess classification performances, we performed a 10-fold, cross-validation procedure using the proposed LDA algorithm. Table 2 shows the results using three different feature vectors: hippocampal shape deformity, amygdalar shape deformity, and the combination of the two shape deformities. We denote each of the three feature vectors as feature 1, feature 2, and feature 3, respectively, for simplification. The sensitivity and the specificity were 95.7% and 68.9% for feature 1, 87.0% and 68.9% for feature 2, and 95.7% and 75.6% for feature 3, respectively (Figure 2(a)). We further assessed the classification accuracy using leave-one-out cross validation, which showed almost the same results for every classifier. Figure 2(c) shows the ROC curve of each classifier. As shown in these results, the performance of our classifier was highest using the whole feature vectors (82.4% accuracy). In the ROC curves, the AUC value for the classifier with feature 3 was 0.8203.

**Table 2:** Classification accuracy for the three different features.

	Feature 1 Hippocampal Shape	Feature 2 Amygdalar Shape	Feature 1+Feature 2
Sensitivity	95.65%	86.96%	95.65%
Specificity	68.89%	68.89%	75.56%
Accuracy	77.94%	75.00%	82.35%

**Figure 3:**
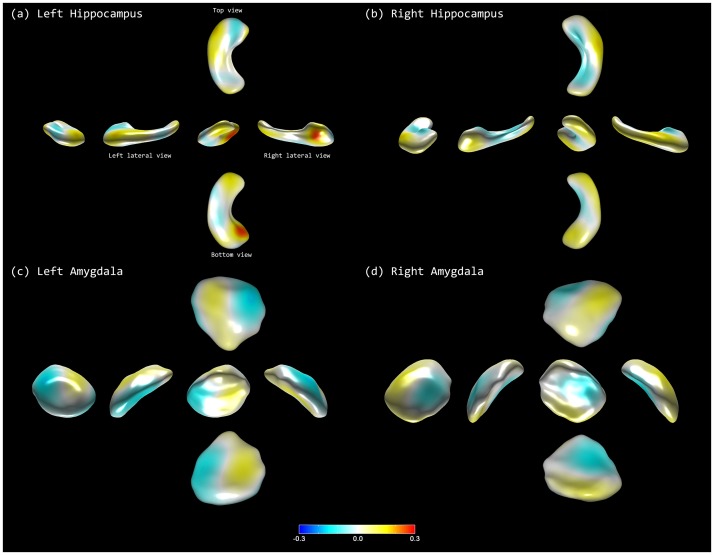
Discriminative regions in classification: (a) left hippocampus, (b) right hippocampus, (c) left amygdala, and (d) right amygdala. Each figure visualizes the LDA axes on the atlas meshes.

We further demonstrated the effectiveness of our incremental classification method. For each of the feature vector, we measured the accuracy of the classifier with respect to the number of patients used in training data. Figure 2(b) depicts the accuracy of every classifier. As shown in the figure, accuracy tended to converge with that of the respective classifier trained with the entire training data as the number of used training patients approached to that of the training patients in the data set. We also extracted the discriminative regions for our classifiers, which provided multivariate patterns contributing to the discriminability between the mixed dementia and pure SVaD groups. Figure 3 depicts the discriminative regions on the atlas surface meshes for our classification. The anterior head, superior portion of body subregions in the hippocampus and the lateral, medial, and central subregions of the amygdala were the discriminative regions.

## Discussion

Using standard T1 weighed MRI, and incremental learning, mixed dementia was distinguished from pure SVaD with 82.4% accuracy. We found that hippocampal shape analysis alone was able to classify mixed dementia from pure SVaD with 77.9% accuracy (95.7% sensitivity and 68.9% specificity). Amygdalar shape analysis was able to classify the two groups with75.0% accuracy (87.0% sensitivity and 68.9% specificity). When both hippocampi and amygdalar shape were incorporated into the analysis, the classification accuracy increased to 82.4% (95.7% sensitivity and 75.6% specificity).

Our individual analysis method has strengths in several aspects. In general, nearby regions within a subcortex have correlated brain functions and are similarly deformed by brain diseases. Our method represents the subcortical deformity data of a subject in terms of spatial frequency components by employing manifold harmonic transform. Thus, the resulting feature vector exploits the spatial coherency of the subcortical deformity data in both learning and classification. The vertex-wise subcortical deformity representation poorly reflects spatial relationship of the feature data since it handles each element of the deformity data independently. On the other hand, a method based on a region-wise representation of the deformity exploits a single value in a region as a feature by averaging all the deformity data in the region, which therefore poorly reflects detailed spatial variation of the deformity data. Our method combines the advantages of the vertex- and region-based methods, while reducing their shortcomings.

It is noteworthy that hippocampal shape alone could discriminate mixed dementia from pure SVaD with 77.9% accuracy. Hippocampi are known to be involved in AD pathologies, and hippocampal volume and shape show significant differences across normal aging, amnestic mild cognitive impairment and AD dementia patients [8], [29]. However, recent studies indicate that white matter lesions are also associated with hippocampal atrophy [30] and that SVaD patients show hippocampal atrophy as well [31], [32]. Furthermore, a recent study from our research group reports that pure SVaD showed significant hippocampal atrophy and shape deformity [33]. In using our method of incremental learning analysis, we found subregions that contributed to discriminating between the two groups. The bilateral anterior head and the medial portion of body of hippocampus (Blue in Figure 3) distinguished mixed dementia from pure SVaD in an opposite direction relative to the bilateral lateral portion of body of hippocampus (yellow in Figure 3). That is, the combination of inward deformities in Blue and outward deformities in Yellow regions, respectively, determines the pattern of shape deformity specific to a certain group in the high-dimensional feature space.

Additional information from the amygdalar shape analysis increased the classification accuracy, both in sensitivity and specificity. According to pathologic studies, the amygdala is one of the brain structures that is involved in the early stages of AD [9]. In addition, MR volumetric studies repeatedly demonstrate amygdalar atrophy in patients with AD dementia [34], [35]. In a study with AD patients, the most affected subregions were the basolateral ventral medial (BLVM) nucleus, which is connected to hippocampus, medial nucleus, and central nucleus [36]. In our classification model, the amygdalar subregions that primarily distinguished mixed dementia from pure SVaD were the right dorsolateral, left dorsomedial, and bilateral ventro-central subregions as colored in yellow in Figure 3, and the right dorsomedial, bilateral ventro-medial subregions as colored in blue in Figure 3. Similarly to the result of hippocampal analysis, different patterns of shape deformity in the blue and yellow colored areas discern mixed dementia from pure SVaD.

In the current study, we hypothesized that AD pathology involve predominantly in the hippocampi and amygdala, which in turn leads to differentiation of mixed dementia from pure SVaD. As shown in figure 3, the colored areas are the regions where it discriminates mixed dementia from pure SVaD. However, strictly speaking, it does not provide information about whether shape deformities in those subregions are more specific to a certain group.

In order to assess classifier performance, we further employed a framework of permutation tests [37]–[39]. Performing 10-fold cross-validation in our experiments, permutation tests were employed to estimate the statistical significance of the classification accuracy. Specifically, we randomly permuted the subject labels of the training data prior to training. The permutation was repeated 10,000 times and the accuracy value of the trained classifier at every permutation was chosen as the statistic, which in turn form a null permutation distribution. The significance level was then estimated over the null distribution by computing the percentile of the accuracy value calculated by the classifier trained on the real subject labels. We conducted this experiment for the classifier using the Feature 3. The resulting p-value was 0.0002 indicating that the classifier learned the relationship between the data and the labels reliably.

This study has several limitations. First PiB-PET may not be sufficiently sensitive to detect soluble amyloid oligomers or diffuse amyloid plaques. Second, we were not able to detect neurofibrillary tangles. Thus, PiB(-) SVaD might not be true ‘pure’ SVaD. Third, the specificity of each classification model was relatively low compared to high sensitivity. It might be related to the fact that shape features specific to pure SVaD are not salient enough to discriminate pure SVaD from mixed dementia. Unbalanced sample sizes could be another important factor for the low specificity. In order to resolve the imbalance between specificity and sensitivity, one may employ classification strategies considering trade-off between them [40]. Fourth, there were differences in age between PiB(+) SVaD and PiB(-) SVaD. It might be related to the fact that age is one of the most important risk factors of amyloid pathology. Therefore, it might be further needed to develop a new classification method that deals with the clinical information. Another limitation of our method is that it requires an empirical selection of two parameters: cut-off frequency 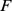 and the dimension of the reduced PCA space. In our study, we used the goodness of fit to determine the cut-off frequency 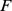: we set the goodness of fit to a bit conservative value and obtained 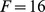. However, the accuracy of a classifier could be improved by selecting optimal 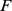 in a classifier-specific manner. A similar argument could be applied to the determination of the reduced PCA space. Nevertheless, this is the first study to provide an individual subject classifier to discriminate mixed dementia from pure SVaD. Our classification method provides a useful tool for classifying mixed dementia from pure SVaD with relatively high accuracy, which has clinical implications for diagnosis and treatment. There are numerous ongoing trials testing amyloid-targeted therapies, whereas no potential disease modifying therapies have emerged for cerebral ischemia. Thus with the use of our classification model, mixed dementia patients may benefit from amyloid-targeted therapies.
